# A rapid qualitative assessment of the impact of the COVID-19 pandemic on a racially/ethnically diverse sample of gay, bisexual, and other men who have sex with men living with HIV in the US South

**DOI:** 10.21203/rs.3.rs-57507/v1

**Published:** 2020-08-12

**Authors:** Scott D. Rhodes, Lilli Mann-Jackson, Jorge Alonzo, Manuel Garcia, Amanda E. Tanner, Benjamin D. Smart, Danielle N. Horridge, Cornelius N. Van Dam, Aimee M. Wilkin

**Affiliations:** Wake Forest School of Medicine; Wake Forest School of Medicine; Wake Forest School of Medicine; Wake Forest School of Medicine; University of North Carolina Greensboro; Wake Forest School of Medicine; Wake Forest School of Medicine; Regional Center for Infectious Diseases, Cone Health; Wake Forest School of Medicine

**Keywords:** COVID-19, HIV, sexual minority, disparities, qualitative

## Abstract

Persons living with HIV (PLWH) may be at increased risk for severe COVID-19-related illness. Our community-based participatory research partnership collected and analyzed semi-structured interview data to understand the early impact of the COVID-19 pandemic on a sample of racially/ethnically diverse gay, bisexual, and other men who have sex with men living with HIV. Fifteen cisgender men participated; their mean age was 28. Six participants were Black/African American, five were Spanish-speaking Latinx, and four were White. Seventeen themes emerged that were categorized into six domains: knowledge and perceptions of COVID-19; COVID-19 information sources and perceptions of trustworthiness; impact of COVID-19 on behaviors, health, and social determinants of health; and general COVID-19-related concerns. Interventions are needed to ensure that PLWH have updated information and adhere to medication regimens, and to reduce the impact of COVID-19 on social isolation, economic stability, healthcare access, and other social determinants of health within this vulnerable population.

## Resumen

Las personas que viven con VIH (PLWH por sus siglas en inglés) pueden tener mayor riesgo de contraer serias enfermedades relacionadas con el COVID-19. Nuestra investigación participativa basada en la comunidad recopiló y analizó datos de entrevistas semiestructuradas para entender el impacto inicial de la pandemia COVID-19 en una muestra de hombres gay, bisexuales y otros hombres que tienen sexo con hombres de diversos grupos étnicos y raciales que viven con VIH. Participaron quince hombres cisgénero con un promedio de edad de 28 años. Seis participantes fueron negros/afroamericanos, cinco latinx hispanohablantes y cuatro blancos. Emergieron diecisiete temas que fueron categorizados en seis ámbitos: conocimiento y percepciones de COVID-19; fuentes de información sobre COVID-19 y percepciones de confiabilidad; impacto de COVID-19 en comportamientos, salud y determinantes sociales de la salud e inquietudes generales relacionadas con COVID-19. Se necesitan intervenciones para garantizar que las personas que viven con VIH tengan información actualizada y cumplan con adherirse a su régimen de tratamiento y reducir el impacto de COVID-19 en lo que respecta a aislamiento social, estabilidad económica, acceso a los servicios de atención médica y otros determinantes sociales de la salud en estas poblaciones vulnerables.

## Introduction

The United States (U.S.) South continues to experience disproportionate HIV rates compared to other regions of the country and has been referred to as the “new” and “latest” U.S. HIV epicenter (1,2). Southern states account for an estimated 51% of new HIV diagnoses in the U.S. each year, despite having only 38% of the county’s overall population. Eight of the 10 states with the highest rates of new HIV diagnoses, and nine of the 10 metropolitan statistical areas with the highest rates, are in the South (2). North Carolina (NC) consistently ranks among these top 10 states for HIV diagnoses (3), and Guilford County, located in the Piedmont Triad region of central NC, has consistently higher rates of HIV than NC and the U.S. overall (4, 5). Guilford County ranks third out of the 100 counties in the state for HIV per 100,000, and its HIV incidence rate is 50% higher than the national rate (6).

Coronavirus disease 2019 (COVID-19) is a new infectious disease caused by the novel severe acute respiratory syndrome coronavirus 2 (SARS-CoV-2), which had not previously been reported in humans (7). Manifestations range from asymptomatic infection to severe complications including pneumonia, acute respiratory distress syndrome, coagulopathies, immune system dysregulation, and death. COVID-19 is highly contagious and has quickly spread globally.(8, 9) The first case of COVID-19 in NC was identified on March 3,2020, and on March 10, NC declared a state of emergency (10). Given the ongoing rise in community-acquired COVID-19 cases in NC, on March 30, a statewide stay-at-home order went into effect that limited activities to those that were considered essential (e.g., health-, safety-, nutrition-, and sanitation-related), banned gatherings of more than ten people, and prioritized social distancing (staying at least 6 feet away from other people) (11). The NC stay-at-home order was eased on May 8, 2020, with a phased re-opening that continued to limit the types of businesses and institutions that could open and the sizes of gatherings.

HIV infection, along with several other immunosuppressing conditions, might increase risk of severe COVID-19-related illness (12). Although the underlying mechanism is not fully understood, this risk for PLWH may be due to a reduction in lymphocytes, immune system dysregulation, or an increased inflammatory response (13). Comorbidities that are becoming increasingly common among PLWH, such as diabetes, chronic kidney disease, chronic obstructive pulmonary disease, and obesity, also increase the risk of developing severe illness from COVID-19 (14).

The burden of these risks may not be carried equally among all PLWH. Communities of color face disproportionate rates of HIV, and Black/African American gay, bisexual, and other men who have sex with men (GBMSM) and Black/African American transgender women have higher rates of HIV than any other group in the US (15, 16). In addition, Black/African American and Latinx PLWH have lower rates of care linkage and retention and are less likely to be virally suppressed than White PLWH (17). In addition, persons of color are also experiencing disparities related to COVID-19. As of June 2020, Black/African American and Native American or Alaska Native persons have COVID-19-related hospitalization rates five times that of non-Hispanic White persons, and Hispanic or Latinx persons have COVID-19 diagnosis rates four times those of non-Hispanic White persons (18). These higher rates may be related to living conditions, work circumstances, and health disparities including insurance and healthcare access as well as higher rates of comorbidities that increase the risk of severe illness from COVID-19, which are shaped by the larger context of systemic racism and inequalities (16, 19). Therefore, racially/ethnically diverse GBMSM living with HIV are a particularly vulnerable population when considering the risk and impact of COVID-19.

Despite the impact of COVID-19 and potential for serious morbidity and mortality, little is known about the pandemic from the perspective of PLWH themselves. Our long-standing community-based participatory research (CBPR) partnership sought to qualitatively explore the impact of the COVID-19 pandemic within a racially/ethnically diverse sample of GBMSM living with HIV.

## Methods

This rapid assessment was conducted by our CBPR partnership in NC that includes racially/ethnically diverse GBMSM living with HIV; representatives from public health departments, HIV service organizations, and clinics that serve PLWH; and academic investigators. This partnership has an established history exploring and intervening on the health-related needs and priorities of vulnerable communities (5, 20-22).

We collected qualitative data from participants who had completed 12 months of participation in and had “graduated” from the *weCare* intervention. *weCare* is an evidence-informed intervention that improves HIV care engagement among racially/ethnically diverse GBMSM and transgender women living with HIV by reducing missed HIV care appointments and increasing viral suppression (21, 23, 24). *weCare* currently is being conducted in partnership with the Regional Center for Infectious Diseases (RCID) in Guilford County, NC (21). RCID is funded by the Ryan White HIV/AIDS Program, Health Resources and Services Administration (HRSA), to serve low-income PLWH who are uninsured or underinsured. RCID provides comprehensive integrated services for PLWH, including HIV primary medical care, case management, bridge counseling, financial assistance, behavioral health care, community outreach nursing, and a dental clinic.

The details of the *weCare* intervention are described elsewhere (21, 23, 24); briefly, *weCare* harnesses social media platforms, including Facebook messenger, text messaging (including through applications or “apps” such as WhatsApp), and messaging through GPS-based mobile apps used for social and sexual networking (e.g., A4A/Radar, badoo, Grindr, Jack’d, and Scruff) to improve linkage to and retention in HIV care among PLWH. The intervention is *targeted* to racially/ethnically diverse GBMSM and transgender women living with HIV, *tailored* to the social media platform preferences of participants, and *personalized* to each participant’s needs (23). Eligibility criteria to participate in *weCare* include: being age 16 or older, identifying as cisgender male or male-to-female transgender, reporting sex with men, and living with HIV. Potential participants are referred to the study by clinic and health department staff. We also advertise the intervention study on Facebook through paid targeted advertisements and on other social media platforms, in a southeastern LGBTQ (lesbian, gay, bisexual, transgender, and queer) newspaper, and through flyers posted within bars, clubs, and coffee shops. Furthermore, we recruit participants through word-of-mouth; enrolled participants are encouraged to share information about the study with others in their social networks. If eligible and interested, participants complete written informed consent procedures and enroll. Participation in the intervention lasts 12 months, after which participants graduate from *weCare*.

Of the graduated *weCare* participants, we randomly selected and contacted 15 of them to complete an interviewer-administered semi-structured individual interview. The abbreviated interview guide is outlined in Table I. Demographic questions included some close-ended items; however, most questions in the interview guide were open-ended to allow participants to describe their experiences, perceptions, and attitudes. A trained bilingual interviewer conducted the interviews in English and Spanish via telephone between April 23 and May 23, 2020.

Interviews were recorded and professionally transcribed. Constant comparison, an approach to grounded theory, was used to analyze data. Constant comparison combines qualitative coding with simultaneous comparison; initial observations are continually refined throughout data collection and analysis (25). Because of the formative nature of this study, we aimed to identify the breadth of experiences, not to quantify them. Analysts coded transcripts and developed matrices to identify similarities and differences within and across interviews. Based on these matrices, each analyst developed preliminary themes. After preliminary themes were developed, the analysts came together via WebEx (a web-conferencing platform) to discuss and reconcile final themes.

Human subject approval and oversight was provided by Wake Forest School of Medicine Institutional Review Board (IRB).

## Results

Fifteen racially/ethnically diverse cisgender men participated; no participants refused to participate. Participant mean age was 28. Six participants were Black/African American, five were Latinx, and four were White; all Latinx participants completed their interviews in Spanish. One participant self-identified as bisexual; all others self-identified as gay. Eleven participants self-reported being virally suppressed while four reported not knowing whether they were virally suppressed.

Seventeen themes emerged from the interviews, categorized into six domains: (1) knowledge and perceptions of COVID-19; (2) COVID-19 information sources and perceptions of trustworthiness; (3) impact of COVID-19 on behaviors; (4) impact of COVID-19 on health; (5) impact of COVID-19 on social determinants of health; and (6) general COVID-19-related concerns. Domains and themes are outlined in Table II.

### 1. Knowledge and Perceptions of COVID-19

#### Knowledge of Transmission and Prevention of COVID-19 Is High

Participants knew a great deal about COVID-19 and its transmission. They were aware of recommended precautions to prevent infection, including wearing a face covering, social distancing, and handwashing. Many were aware of the NO stay-at-home order that was in effect at the time of the interview and reported adhering to the order. For example, a participant reported,

*It’s contagious. It affects the respiratory system. It can be fatal in some cases; but some people get through. It’s important to take precautions and stay at home like the governor says*. (Participant [P]13, Black/African American, 26 years old)

Another participant noted, “*Es un virus muy contagioso, que se transmite de una persona enferma si está muy cerca cuando tose. También, si la persona enferma toca algo, y la persona sana toca la misma superficie, se puede contagiar*.”[‘It is a very contagious virus that is transmitted from a sick person if they are very close when they cough. Also, if the sick person touches something, and the healthy person touches the same surface, it can be contagious.”] (P4, Latinx, 35 years old)

#### COVID-19 Is Perceived as Serious, and Participants Perceive Themselves to be Susceptible

Participants reported that COVID-19 was serious and that those with compromised immune systems were especially at risk. They were worried about their own increased risks as PLWH and noted that hearing about others who had similar characteristics to them being seriously affected also increased their concerns. A participant stated,

*Creo que es muy serio porque nunca en mi vida había visto algo así Afecta a las personas que tienen el sistema inmunológico comprometido como personas con diabetes o con problemas respiratorios, así que es muy serio. Uno piensa que porque uno es joven no le va a pasar, pero estamos viendo casos de jóvenes que han muerto por este virus y eso lo hace más grave para mí*. [I think it is very serious because I have never seen anything like this in my life. It affects people who have compromised immune systems like people with diabetes or respiratory problems, so it's very serious. You think that because you are young it will not happen to you, but we are seeing cases of young people who have died from this virus and that makes it more serious for me.] (P3, Latinx, 28 years old)

Another participant said, “ *The coronavirus is making me super cautious, I am just afraid to contract it so I think about it a lot, but I try to stay optimistic about the entire situation*.” (P14, Black/African American, 27 years old)

Participants also reported worrying about the well-being of friends and family members, particularly those who were older, had comorbidities, or had other risk factors for developing serious illness from COVID-19. A participant noted, “*Pues, estoy muy preocupado por toda mi familia y mi círculo porque no solo me puede afecta ra mí, sino a toda mi familia también*.”["Well, I am very concerned about my whole family and my circle because it can affect not only me, but my entire family as well."] (P5, Latinx, 25 years old)

#### Confusion Exists from Conflicting Information about COVID-19

Participants found available and prevailing information regarding the transmission and prevention of COVID-19 to be conflicting. However, some reported verifying information by referring to several different sources. A participant shared,

*Everyone is saying different things. They give different timeframes about symptoms. It gets very confusing. So I look for similarities across different [sources of] information that I receive. But it is hard to know what is true, what are assumptions, and what are lies*. (P7, Black/African American, 23 years old)

### 2. COVID-19 Information Sources and Perceptions of Trustworthiness

#### Information About COVID-19 Is Obtained From Social Media, the Internet, Television, the Workplace, and Word-of-Mouth

Social media (e.g., Facebook), the internet (e.g., CDC website), and television (e.g., English- and Spanish-language national and local news) were noted as places that participants obtained information about COVID-19. Participants also reported that they received information from their workplaces, as guidelines and regulations changed and thus required changes to how work was performed. For example, participants who worked in restaurants noted that they learned about transmission, prevention, and the current state of disease burden from restaurant owners and managers. In addition, participants described friends and family as sources of information about COVID-19. A participant reported, “*I get info on TV, and I did some research on the internet, read some articles, and heard some through word-of-mouth, like during a meeting at work when we go over protocols and changes that are going into effect because of the coronavirus*.”(P10, White, 28 years old)

#### The President of the United States Is Not a Trusted Source of COVID-19 Information

Participants reported having little trust in what U.S. President Donald Trump communicated about the pandemic. They did not trust his assessment of the potential impact of the pandemic, his advice regarding how to prevent COVID-19 transmission, or his assertions regarding its treatment. A participant noted,

*I don’t trust what the president of the United States says, especially because we have people like Dr. Fauci who immediately say ‘that’s actually not the case.’ Also there is the World Health Organization that is saying that what the president is recommending is not okay. Other doctors working on this are saying that the president is just making things up as he goes. I don’t trust him*. (P7, Black/African American, 23 years old)

Another participant agreed saying, “*I honestly do not trust anything that the president says*.” (P14, Black/African American, 27 years old)

A third participant stated, “*No confío mucho porque creo que no le tomaron mucha importancia cuando empezó y por eso estamos con estos números tan altos*.”["I do not trust much, because I think they did not take it seriously when it started and that is why we have these high numbers."] (P4, Latinx, 35 years old)

#### Providers Are a Highly Trusted Source of COVID-19 Information

Healthcare providers, including HIV providers, were identified in particular as providing timely and trustworthy information about COVID-19. A participant stated, “*I trust my doctor, especially because I believe they have the best interest at heart and they are the people on the frontline. They are telling us what they understand and what they are seeing from their patients*.” (P7, Black/African American, 23 years old)

### 3. Impact of COVID-19 on Behaviors

#### Participants are Taking Action to Reduce Their Risks

Participants reported staying home, maintaining social distancing, and cleaning with disinfectants and sanitizers to prevent contracting COVID-19. A participant commented, “*I have not been out. Today is actually the first time I went out to go to the store. No hugging anyone; I try to stay away from people as much possible or at least six feet*.” (P6, Black/African American, 21 years old) This commitment to prevention was noted by other participants. For instance, a participant stated, “*I have been extremely social distancing, I, in fact; haven’t left my house; some of my friends do the grocery shopping for me and leave my groceries by the door, I bring them inside, sanitize them, and throw the bags out*.” (P7, Black/African American, 23 years old)

Further, another participant noted, “*Guardo mi distancia entre las personas cuando salgo a la tienda, uso desinfectante de alcohol en mis manos, mascarilla cuando salgo afuera, y limpio con desinfectante las áreas que más toco como el refrigerador, las llaves, mi tarjeta de debito, y las mancillas de la puerta*.” [“I keep my distance from people when I go out to the store, use alcohol disinfectant on my hands, a mask when I go outside, and clean the areas I touch the most with disinfectant, such as the refrigerator, keys, my debit card, and the door handles.”] (P3, Latinx, 28 years old)

#### COVID-19 is Having a Mixed Impact on Health Behaviors

Participants noted that worries and fears related to COVID-19 and behavioral changes that they are making to stay safe are affecting them in both negative and positive ways. A participant noted that he is sleeping less because he is worried about his health, the health and well-being of his friends and family, and the long-term impact of the pandemic locally and globally, reporting, “*My sleeping schedule has been changing a lot. It is hard to sleep 8 hours as I used to*.” (P8, Black/African American, 21 years old). Another participant commented, “*I am working out less to avoid public settings*.” (P9, White, 23 years old)

However, some participants reported positive changes they had made in their health behaviors as a result of staying home and other adaptations related to COVID-19 prevention. A participant noted, “*I stopped drinking alcohol because I figure that I have bad habits when I am at home alone; because in the first week or so of the stay home order for the coronavirus, I was drinking a lot and that kind of hurt me*.” (P11, White, 25 years old). Further, another participant shared, “*I actually started meditating in the mornings so I can have a good set of mind about all this. And I have been spending more time in the backyard and gardening more than I did before*.”(P15, White, 27 year old)

#### Use of Social Media for Socialization and Support Has Increased

Before the initiation of the COVID-19 pandemic, participants were already communicating frequently through social media platforms such as Facebook, texting, and GPS-based mobile apps. After the pandemic began, however, participants reported increased use of these types of social media platforms. A participant noted, “*I use Facebook much more now because it is the only way we have to communicate with other people*.” (P12, Black/African American, 21 years old)

Use of other technology for communication, such as video calls, also increased, as another participant added,

“*I don’t see my friends and family much now, and if I do, we try to FaceTime on the iPhone or call each other on Facebook to see them on video. We talk on the phone to catch up. That is the only way we do it to be safe and cautious*.” (P10, White, 23 years old)

### 4. Impact of COVID-19 on Health

#### Feelings of Isolation, Hopelessness, and Worry Are Common

Participants reported that their mental health was profoundly affected by COVID-19 and the necessary precautions required to reduce risks of exposure. A participant noted, “*The changes make me feel hopeless and sad. I am just realizing that everyone wants things to go back to normal, but I think a lot of things will change, and lots of polices that are in place will change, like in restaurants and bars. It will never be normal*.” (P7, Black/African American, 23 years old) Another participant reported, “*I do feel alone, and it kind of reminds me of when I learned that I had HIV*.” (P15, White, 27 years old)

#### Accessing Medical Care is More Difficult

Participants reported that accessing medical care had become more difficult in the context of COVID-19. Though as of the time of data collection participants had not experienced interruptions in their HIV care, some had not been able to get other needed care. As a participant reported, “*It [COVID-19]just makes things harder. I was not able to go to the dentist to take out my wisdom teeth because they do not do anything that is not a priority*.”(P6, Black/African American, 21 years old). Participants had also had challenges with virtual appointments (e.g., telemedicine). For example, a participant shared, “*For the follow-up appointment I had to see the doctor through Zoom, but he couldn’t really see me and that made things more challenging, especially because the technology wasn’t really working. Things are more complicated now*.” (P14, Black/African American, 27 years old)

#### Medication Adherence Is Difficult Due to the Interruption of Routines

Overall, participants reported obtaining HIV medications as needed since the initiation of the pandemic. However, they did report that adhering to medication regimens had become more difficult. A participant shared, “*It’s been easy to get the medications because the pharmacy sends them, but it has been challenging to take them as I should because I had a routine before. I used to take my medication every day at work, but since I am working from home, I can’t follow the same routine*.” (P15, White, 27 years old)

### 5. Impact of COVID-19 on Social Determinants of Health

#### Educational Opportunities and Jobs Were Lost

Participants reported multiple ways in which COVID-19 affected them through social determinants of health. First, some participants noted that they had lost educational opportunities. A participant reported, “*I was supposed to have an internship this summer and make money. It’s been very stressful, and I am not having a good summer*” (P6, Black/African American, 21 year old). Another participant described losing his job, “*I have been laid off from work till further notice, so I have not been working. So it is affecting me that way*.” (P10, White, 28 years old)

Participants felt the financial impact of job losses on other social determinants, such as housing stability. A participant shared, “*Ya empiezo a tener dificultad; no sé cómo voy a hacer este mes para pagar mi renta*.”["I am already having difficulty; I don’t know what I am going to do this month to pay my rent.”] (P5, Latinx, 25 years old)

#### Workplace Exposure Is a Worry

Participants who had jobs and were able to continue working during the stay-at-home order felt fortunate to be able to maintain an income. However, they also worried about exposure to COVID-19 when fulfilling roles as essential workers. A participant noted, “*I think what makes it challenging is that I work in the public and interact with people every day and I am not sure where they have been and if they have COVID-19 or not. I just think that interacting with people who might have it on a daily basis makes it hard*.” (P14, Black/African American, 27 years old)

#### In-Person Social Support Has Been Sacrificed

Participants reported a reduction in in-person social support. A participant noted, “*It* i*s affecting my interactions with other people; I do not see my family and friends anymore*.” (P9, White, 23 years old). Another remarked on his loneliness saying, “*There are times when I think, if I had a boyfriend or a roommate, someone that at least can stay in quarantine with me, that will be better. I mean, I have a cat, but it is just hard sometimes*.” (P10, White, 28 years old)

### 6. General COVID-19-Related Concerns

#### The Economy and its Impact on Self, Families, and Friends Are Concerns

Participants also reported worrying about the long-term implications of the pandemic for local, regional, and global economies. As a participant shared,

*My biggest concern is [that] the way that it is happening here will have a devastating impact on the economy, just because it’s been so bad. I have such a big fear that this will reach areas of underdeveloped counties where the health system is even weaker than ours, and they will not be able to handle that. In addition, I think that when the economy is not doing well in America, the whole world economy falls after that*. (P7, Black/African American, 23 years old)

#### States May Be “Opening Up” Too Quickly

Participants also worried that states may be re-opening businesses and institutions before the COVID-19 pandemic was under control, which could lead to more transmission. As a participant noted, “*They are leaving it to the states to reopen or not, so I am worried about its [COVID-19] being spread*.” (P9, White, 23 years old)

## Discussion

PLWH may be at increased risk for severe COVID-19-related illness. In this qualitative study of a sample of racially/ethnically diverse GBMSM living with HIV, we identified 17 themes that we grouped into six domains. Several findings deserve further attention. First, participants had high levels of knowledge about the transmission and prevention of COVID-19. While participants reported some confusion about conflicting and emerging information related to COVID-19, they utilized multiple and credible sources to obtain information about the pandemic and risk reduction. This finding could reflect, in part, their past participation in the *weCare* intervention, which improves health literacy by helping participants identify, evaluate, and use online sources of health information, including CDC and HRSA websites (21, 23, 24). Thus, despite our sample knowing quite a bit about COVID-19, many PLWH may need support in identifying and accessing correct and updated information to manage their health in light of the dissemination of conflicting information regarding COVID-19.

Further, HIV healthcare providers were identified as highly trusted sources of information about COVID-19. Providers must make time to assess COVID-19-related information needs, correct misinformation, and support risk reduction among their patients living with HIV. Participants did not, however, trust information provided by U.S. president. Participants distinguished between the veracity of information about COVID-19 provided by the U.S. president and information about COVID-19 provided by other government leaders such as Dr. Anthony Fauci, the Director of the National Institute of Allergy and Infectious Diseases (NIAID) and a member of the White House Coronavirus Task Force.

It is important to note that this study was conducted early in the COVID-19 pandemic, and many participants had not yet navigated medical care, such as a routine office visit for HIV care, since the implementation of changes in care delivery related to COVID-19. However, some participants reported having non-urgent healthcare visits canceled or postponed, and those participants who had obtained care reported not being satisfied with telemedicine appointments. COVID-19 has required acceleration of telemedicine, and much is being learned about how best to provide care using this distance platform (26). As telemedicine becomes more widespread, concerns about confidentiality, access to and quality of care, reliable internet access, and health disparities must be considered. In our research with GBMSM living with HIV in the U.S. South, we have found that most own or have access to smartphones; however, most do not have access to desktop computers or laptops at home. Thus, they may not have the requisite technology and/or a private place to participate in telemedicine visits that utilize a video component (21, 23, 24, 27, 28). Transition to telemedicine could potentially reduce HIV care engagement and viral suppression and increase disparities among some of the most vulnerable populations living with HIV. Current efforts to improve telemedicine technology and the integration of telemedicine into clinic settings should include targeted efforts to meet the needs of vulnerable populations, including PLWH.

Moreover, while COVID-19 did not seem to affect access to HIV medications in this study, the impact of the pandemic affected adherence to medical regimens. Interventions may be needed to help PLWH strategize how and when they can take their HIV medications given interruption to their routines due to COVID-19 (e.g., changes in job and school schedules).

PLWH may be particularly affected by COVID-19 because they may already be experiencing stigma and isolation related to living with HIV (9). Participants noted that living through the COVID-19 pandemic felt like when they first learned of their HIV status, with similar feelings of loneliness and having no one to turn to. They reported limiting their interactions with others to reduce their risk of exposure; however, they subsequently reported feeling alone, isolated, and in need of social support. These feelings can lead to hopelessness and depression and negatively affect medication adherence and health outcomes (29). To address these challenges, interventions may be needed to bolster social support among PLWH while maintaining social distancing. Given that participants reported using social media at increasing levels since the initiation of the pandemic, approaches that leverage these platforms, such as those used in *weCare*, may hold particular promise in this context.

Participants also noted that the COVID-19 pandemic resulted in missed educational opportunities, job loss, and financial hardship. Moreover, their worries were broad; they expressed concerns about themselves as well as others who they care about and the impact of the pandemic on the global economy. Interventions are needed to address the varied and pervasive harms, health-related and otherwise, caused by the COVID-19 pandemic on PLWH, and also the underlying social determinants of health that can make PLWH more vulnerable to these harms.

### Limitations

It is important to note that participants in this study do not reflect the entire population of PLWH or PLWH in the U.S. South. Participants represented a unique sample and may have benefited from their participation in *weCare*. Further studies are needed with participants that have not participated in such an intervention. We also note the small sample size; however, we purposefully recruited a racial/ethnically diverse sample and terminated data collection when we reached saturation. We also note that we wanted to better understand the breadth of experiences of PLWH; future studies should focus on quantifying the experiences identified and developing targeted interventions for PLWH based on these findings. This study was designed to be a first, early step.

## Conclusion

This study tapped into a small group of racially/ethnically diverse GBMSM living with HIV. It lays the foundation for future research exploring both the immediate and long-term impacts of the COVID-19 pandemic among GBMSM living with HIV. Additionally, there is profound need for novel interventions to address the impact of the COVID-19 pandemic on social isolation, economic stability, access to health care, and other social determinants of health for racially/ethnically diverse GBMSM living with HIV. Because increased age is a risk factor for severe illness from COVID-19, and nearly half the PLWH in the U.S. are over 50 (30), further indicating the potential of severe COVID-19-related illness among this population, there is also need for targeted research to understand the impact of the pandemic on older PLWH and interventions to support older PLWH’s needs related to COVID-19.

These data were collected within the first three months after the first COVID-19 case was reported in NO and provide important insights about the impact of this rapidly emerging public health issue. Since then, new developments have occurred, including a statewide mandate requiring the use of face coverings in public places that went in to effect in NC on June 24, 2020. At the same time, while participants reported engaging in prevention behaviors initially, their adherence with social distancing, face coverings, and use of disinfectants and sanitizers may decrease over time as fatigue sets in. It will be important to build on these early findings within the continually changing context in terms of COVID-19 infection rates, economic impacts, and state and federal government responses.

## Figures and Tables

**Table I.** Semi-structured interview guide: Abbreviated content

Demographics
Awareness and knowledge of COVID-19
Perceived severity to COVID-19
Perceived susceptibility of infection: Self and family
Preventive actions to reduce one’s risks
Sources of information about coronavirus/COVID-19
Provider trust regarding coronavirus
Trust of U.S. government
Use of social media in era of coronavirus
How coronavirus has affected use of healthcare services and HIV medications
How coronavirus has affected social determinants of health, such as employment, housing, food access, transportation, social support
Mental health
Biggest worries related to COVID-19

**Table II.** Early impact of the COVID-19 pandemic on a sample of racially/ethnically diverse gay, bisexual, and other men who have sex with men living with HIV: Domains and themes

**(1) Knowledge and Perceptions of COVID-19**
Knowledge of Transmission and Prevention of COVID-19 is High
COVID-19 Is Perceived as Serious, and Participants Perceive Themselves to be Susceptible
Confusion Exists from Conflicting Information about COVID-19
**(2) COVID-19 Information Sources and Perceptions of Trustworthiness**
Information about COVID-19 Is Obtained From Social Media, the Internet, Television, the Workplace, and Word-of-Mouth
The President of the United States Is Not a Trusted Source of COVID-19 Information
Providers Are a Highly Trusted Source of COVID-19 Information
**(3) Impact of COVID-19 on Behaviors**
Participants are Taking Action to Reduce Their Risks
COVID-19 is Having a Mixed Impact on Health Behaviors
Use of Social Media for Socialization and Support Has Increased
**(4) Impact of COVID-19 on Health**
Feelings of Isolation, Hopelessness, and Worry Are Common
Accessing Medical Care is More Difficult
Medication Adherence Is Difficult Due to the Interruption of Routines
**(5) Impact of COVID-19 on Social Determinants of Health**
Educational Opportunities and Jobs Have Been Lost
Workplace Exposure Is a Worry
In-Person Social Support Has Been Sacrificed
**(6) General COVID-19-Related Concerns**
The Economy and its Impact on Self, Families, and Friends Are Concerns
States May Be “Opening Up” Too Quickly
